# A Novel Homozygous Missense Variant of *PIGT* Related to Multiple Congenital Anomalies-Hypotonia Seizures Syndrome 3 with Elevated of Serum ALP Level in a Thai Newborn Patient

**DOI:** 10.3390/ijms26062790

**Published:** 2025-03-20

**Authors:** Jeerawan Klangjorhor, Natrujee Wiwattanadittakul, Thanapak Jaimalai, Patcharawadee Thongkumkoon, Pitiporn Noisagul, Ratchadaporn Khiaomai, Nutnicha Sirikaew, Nonthanan Moonsan, Arnat Pasena, Pathacha Suksakit, Pimpisa Teeyakasem, Parunya Chaiyawat, Maliwan Tengsujaritkul

**Affiliations:** 1 Center of Multidisciplinary Technology for Advanced Medicine (CMUTEAM), Faculty of Medicine, Chiang Mai University, Chiang Mai 50200, Thailand; jeerawan.klangjorhor@cmu.ac.th (J.K.); thanapak.jaim@cmu.ac.th (T.J.); patcharawadee.t@cmu.ac.th (P.T.); pitiporn.noi@cmu.ac.th (P.N.); ratchadaporn.kh@cmu.ac.th (R.K.); nutnicha_siri@cmu.ac.th (N.S.); arnat.pas@cmu.ac.th (A.P.); pathacha.s@cmu.ac.th (P.S.); parunya.chaiyawat@cmu.ac.th (P.C.); 2 Office of Research Administration, Chiang Mai University, Chiang Mai 50200, Thailand; 3 Musculoskeletal Science and Translational Research (MSTR), Faculty of Medicine, Chiang Mai University, Chiang Mai 50200, Thailand; pimpisa.t@cmu.ac.th; 4 Department of Pediatrics, Faculty of Medicine, Chiang Mai University, Chiang Mai 50200, Thailand; nattarujee.w@cmu.ac.th (N.W.); nontanun.moonsan@cmu.ac.th (N.M.)

**Keywords:** neonatal hypotonia, multiple congenital anomalies-hypotonia seizures syndrome 3 (MCAHS3), PIGT, whole exome sequencing

## Abstract

Phosphatidylinositol glycan class T (PIGT) is part of the glycosylphosphatidylinositol transamidase (GPI-TA) complex, crucial for various cell functions. Biallelic pathogenic variants in *PIGT* are associated with Multiple Congenital Anomalies-Hypotonia Seizures Syndrome 3 (MCAHS3), a rare neonatal hypotonia syndrome characterized by dysmorphic features and seizures. Diagnosing neonatal hypotonia, which has diverse congenital and acquired causes, is challenging, particularly in syndromic monogenic cases. Next-generation sequencing is essential for accurate diagnosis. This study reports a term newborn with hypotonia, dysmorphic features, seizures, and severe skeletal issues, including a humeral fracture at birth, consistent with MCAHS3. Trio whole exome sequencing (WES) analysis revealed a novel homozygous missense variant in *PIGT*, expanding the clinical spectrum of MCAHS3 and marking the first such case in the Thai population. The identified c.257A>G (p.His86Arg) variant manifests a severe MCAHS3 phenotype, as evidenced by reduced CD59 expression in western blot analysis, indicating impaired GPI-AP synthesis. Computational predictions suggest this mutation causes protein instability, potentially affecting GPI anchor attachment. While alkaline phosphatase (ALP), a GPI-AP crucial for skeletal mineralization, was elevated in this case, suggesting a late-stage GPI synthesis defect. The His86Arg mutation in PIGT may disrupt GPI-TA function, hindering proper protein attachment and leading to cleaved protein secretion. Further functional studies are needed to elucidate the impact of this mutation on PIGT function and MCAHS3 phenotypes.

## 1. Introduction

In eukaryotes, numerous proteins require specific anchor molecules for their expression and function on the plasma membrane. Glycosylphosphatidylinositol-anchored proteins (GPI-APs) are proteins attached to the plasma membrane via glycosylphosphatidylinositol (GPI), which acts as a glycolipid anchor. There are more than 150 GPI-APs in humans, playing crucial roles in various cell functions, including embryogenesis, neurogenesis, cell signaling, adhesion, receptor activity, complement regulation, transcellular transport, and enzymatic activities [1]. The biogenesis of GPI-APs is a conserved post-translational process that includes two parts: the synthesis of GPI anchors and their attachment to proteins, both of which occur in the endoplasmic reticulum (ER) and Golgi apparatus. At least 31 genes encode proteins that mediate GPI-AP synthesis, and pathogenic variants in 22 of these genes have been identified as related to human diseases so far [2,3].

The GPI transamidase (GPI-TA) complex facilitates the attachment of GPI anchors to proteins in the ER. The GPI-TA is a heteropentameric complex composed of five protein subunits: PIGK, Gaa1, PIGS, PIGU, and PIGT. The *PIGT* gene encodes the phosphatidylinositol glycan class T (PIGT). Biallelic pathogenic variants in *PIGT* are associated with Multiple Congenital Anomalies-Hypotonia Seizures Syndrome 3 (MCAHS3, OMIM 615398). MCAHS3 is an autosomal recessive disorder characterized by multiple dysmorphic features related to hypotonia syndrome. The clinical manifestations include craniofacial dysmorphic features such as a high forehead, frontal bossing, bitemporal narrowing, depressed nasal bridge, and high arched palate, along with seizures and various types of abnormal epileptiform patterns on electroencephalogram (EEG) due to cerebral and cerebellar atrophy, as well as moderate to severe developmental delay. Kvarnung et al. first reported a homozygous missense variant in *PIGT* in four patients with MCAHS3 from consanguineous Turkish kindred in 2013 [4]. About 40 cases of MCAHS3 have been reported to date.

In the Thai population, this disease has never been previously reported. Here, we describe the first Thai patient with MCAHS3, characterized by dysmorphic features, hypotonia, and elevated serum alkaline phosphatase (ALP) level caused by a novel homozygous missense variant in the *PIGT* gene. We examined the effect of this novel variant on PIGT protein structure and function to determine its relationship to patient phenotypes.

## 2. Results

### 2.1. Case Report

**Figure 1:**
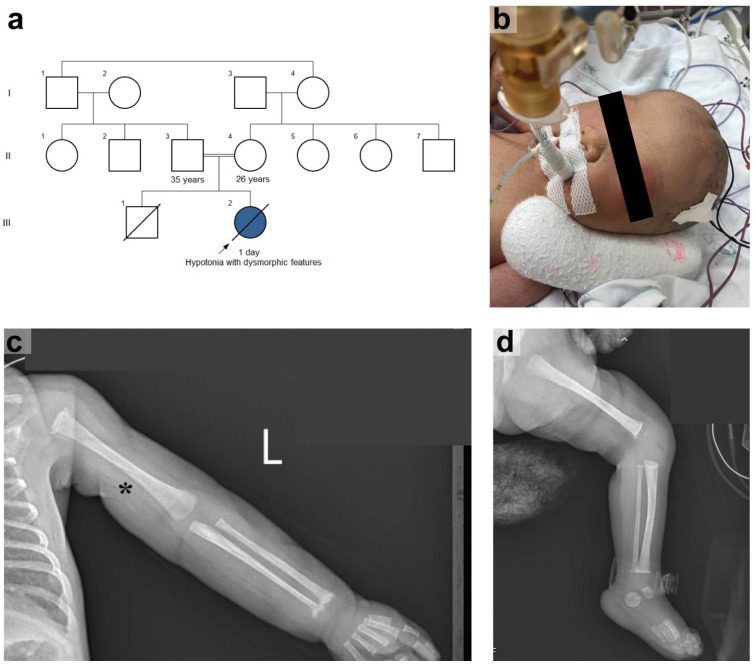
Pedigree and clinical manifestations of the patient. (**a**) The pedigree shows a consanguineous marriage, and each generation is identified by a Roman numeral (I, II, III). (**b**) Ocular hypertelorism, a flat nasal bridge, and low-set ears were observed. (**c**,**d**) The radiographic study shows an incidental left (L) humeral fracture, indicated by an asterisk (*), osteopenia, widening of the metaphysis of the proximal humerus and distal radii, and ossified distal femoral epiphyses.

A term female newborn was born via cesarean section due to breech presentation to first-cousin parents. Her mother had previously experienced a spontaneous abortion at six weeks during her first pregnancy (Figure 1a). In this pregnancy, there was no antenatal complication. At birth, the infant exhibited irregular breathing and poor muscle tone. The physician at the primary hospital decided to intubate her, administer empirical antibiotics, and refer her to our hospital. Upon arrival at our hospital, a physical examination revealed normal growth parameters: a birth weight of 2885 g (50th percentile), a length of 48 cm (50th percentile), and a head circumference of 36 cm (50th–90th percentile). Dysmorphic features include ocular hypertelorism, flat nasal bridge, low-set ears, and bilateral hand contractures were noticed (Figure 1b). The neurological examination showed flaccid tone, no spontaneous movement, pupils 2 mm reactive to light bilaterally, weak sucking reflex, negative Moro and grasping reflexes, and 1+ deep tendon reflexes in all limbs. Other findings, including hepatosplenomegaly and skin rashes, were not detected.

Laboratory tests were performed to determine the etiology of neonatal hypotonia. Complete blood count showed normal hemoglobin and hematocrit (Hb 16.3 g/dL, Hct 49.6%), elevated white blood cell count (24,580 cells/mm^3^) with neutrophil predominance (neutrophils 84%, lymphocytes 9.6%, monocytes 5.6%, basophils 0.3%, eosinophils 0.5%), and normal platelets (200,000 cells/mm^3^). The sepsis screening results were within the normal range (I:T ratio 0.15, mESR 3 mm, CRP < 3.41). Cerebrospinal fluid (CSF) profile showed no abnormalities (RBC 2 cells, WBC 1 cell, glucose 53 mg/dL [POCT 82 mg/dL; ratio 0.64], protein 130 mg/dL). Hemoculture and CSF culture reported no bacterial growth. Blood chemistry indicated mild metabolic acidosis with Na 135 mmol/L (normal range 136–145 mmol/L), K 3.9 mmol/L (normal range 3.4–4.5 mmol/L), Cl 104 mmol/L (normal range 98–107 mmol/L), CO_2_ 18 mmol/L (normal range 22–29 mmol/L), and mildly elevated ammonia at 93.3 µg/dL (normal range 18.7–86.9 µg/dL). Comprehensive metabolic tests and plasma amino acid analysis were normal. CSF/plasma glycine ratio was 0.01, excluding inborn errors of metabolism and non-ketotic hyperglycinemia (NKH). Chromosome study was 46,XX.

An incidental humeral fracture was found on chest X-ray. A bone survey showed osteopenia, widening of the metaphysis of the proximal humerus and distal radii, and ossified distal femoral epiphyses (Figure 1c,d). Calcium and phosphate levels were normal (Ca 8.7 md/dL, normal range 8.6–10.2 mg/dl); P 4.3 md/dL, normal range 2.5–4.5 md/dL). Alkaline phosphatase (ALP) levels were consistently high (111–348 U/L, normal range 35–104 U/L). Vitamin D levels were normal (34.12 ng/mL, normal range > 30 ng/mL), and ricket was excluded.

**Figure 2:**
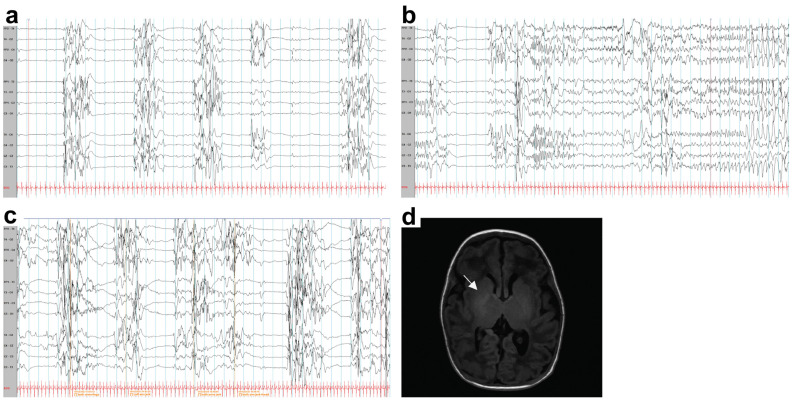
Electroencephalogram (EEG) and brain magnetic resonance image (MRI). (**a**) EEG at day of life (DOL) 13 (time constant 0.3, high-frequency filter 70, and 50Hz notch filter) shows a suppression–burst pattern. (**b**) Electrographic seizures and (**c**) EEG at DOL 75 show a suppression–burst pattern with time-locked myoclonic jerks during the bursts, the asterisks (*) indicate the body parts; both arms and legs, left arm, both arms, both arms and head (time constant 0.3, high-frequency filter 70, and 50 Hz notch filter). (**d**) Brain MRI (T1 FLAIR) shows a loss of T1 hypersignal along the posterior limbs of the bilateral internal capsules with patchy T1 hyperintense signals in the bilateral thalami and lentiform nuclei (arrow).

The bedside video EEG was performed to assess the degree of encephalopathy and revealed a suppression–burst pattern both while awake and asleep. Numerous electrographic seizures were observed (Figure 2a–c). Subsequently, the patient developed erratic myoclonic jerks. Antiseizure medications, including levetiracetam followed by phenobarbital, were initiated. While there has been some improvement in seizure control, there has been no change in the EEG background. A brain MRI on day of life (DOL) 37 showed a loss of T1-hyperintense signal along the posterior limb of the bilateral internal capsules, with patchy T1-hyperintense signals in the bilateral thalami and lentiform nuclei, which is compatible with hypoxic-ischemic encephalopathy (Figure 2d). The periventricular calcification or hyperintensity at the temporal lobes and brainstem were not identified. However, the clinical severity of the patient is out of proportion to the Apgar score and brain imaging findings, prompting further investigation.

We excluded potential causes of neonatal hypotonia, such as perinatal asphyxia, brain malformations, chromosomal abnormalities, congenital infection, and metabolic disorders. The remaining etiologies might be from monogenic disorders, leading us to perform trio–whole exome sequencing. Despite continued ventilatory support, the patient passed away from respiratory failure at the age of five months.

### 2.2. Germline Mutation Analysis

A homozygous c.257A>G (p.His86Arg) variant in *PIGT* was identified in this patient through trio–WES analysis. Sequence data quality assessment confirmed the presence of this variant. Briefly, in the proband, this variant was found in a homozygous state with a total read depth of 57 reads (reference allele A = 0 reads [0%], alternative allele G = 57 reads [variant allele fraction (VAF) = 100%]), including 32 forward read strands and 25 reverse read strands. The genotype quality (GQX) was 99, and the variant quality score was 1765.06. In her parents, this variant was found in a heterozygous state. In the father, the total read depth was 95 reads (reference allele A = 34 reads [35.79%], alternative allele G = 61 reads [VAF = 64.21%]), with 34 forward read strands, and 27 reverse read strands, GQX = 99, and a variant quality score of 1634.64. In the mother, the total read depth was 91 reads (reference allele A = 41 reads [45.05%], alternative allele G = 50 reads [VAF = 54.95%]), with 32 forward read strands, and 25 reverse read strands, GQX = 99, and a variant quality score of 1126.64. The BAM files also confirmed the presence of the *PIGT*: c.257A>G (p.His86Arg) variant (Figure S1).

**Figure 3:**
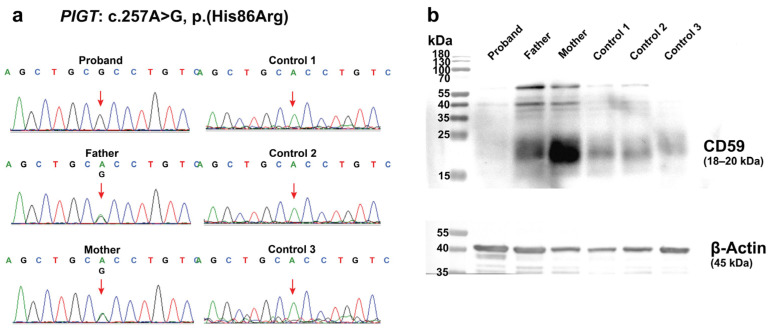
Sanger sequencing data and CD59 GPI-AP expression in mononuclear cells. (**a**) Sanger sequencing of exon 2 of *PIGT* confirmed the presence of the c.257A>G variant in a homozygous state in the proband and in a heterozygous state in the healthy parents. The variant was not present in the wild-type controls. The red arrows indicate c.257 of the *PIGT* gene. (**b**) Western blotting demonstrated lower expression of CD59 in mononuclear cells of the proband compared to those of the parents and wild-type controls.

The patient’s healthy parents are heterozygous carriers of this variant, which was confirmed by Sanger sequencing (Figure 3a). This variant is in exon 2 out of 12 exons. The mutation replaces histidine with arginine, both of which are positively charged amino acids. This variant has been reported in a heterozygous form with a very low frequency in gnomAD (<0.01%) and has not been found in Thai exome databases. Computational prediction tools consistently suggest a deleterious effect on the gene (REVEL = 0.7, deleterious; BayesDel = 0.2, deleterious; M-CAP = 0.036, possibly pathogenic; and CADD = 25.1, top 1% of the most potentially harmful variants). However, this variant has not been previously reported in affected individuals. The current evidence is insufficient to conclusively link this variant to the disease. According to ACMG/AMP guidelines, this variant is classified as a variant of uncertain significance.

### 2.3. Effects of the c.257A>G on PIGT Function Demonstrated by GPI-AP Expression Analysis

Due to sample limitations, only CD59 expression was analyzed, which may not fully represent the global impact of the PIGT mutation on GPI-APs. CD59 protein expression in PBMCs from the patient was compared with that in PBMCs from the parents and age-matched wild-type controls (control 1 and 3 = 1 day old; control 2 = 4 months old). Western blotting revealed that CD59 levels in the patient’s mononuclear cells were lower compared to those in the parents and controls (Figure 3b). This data suggests that the homozygous missense variant leads to impaired GPI-APs in the patient.

### 2.4. The Intramolecular Interactions Among Amino Acids at the Site of Alteration

**Figure 4:**
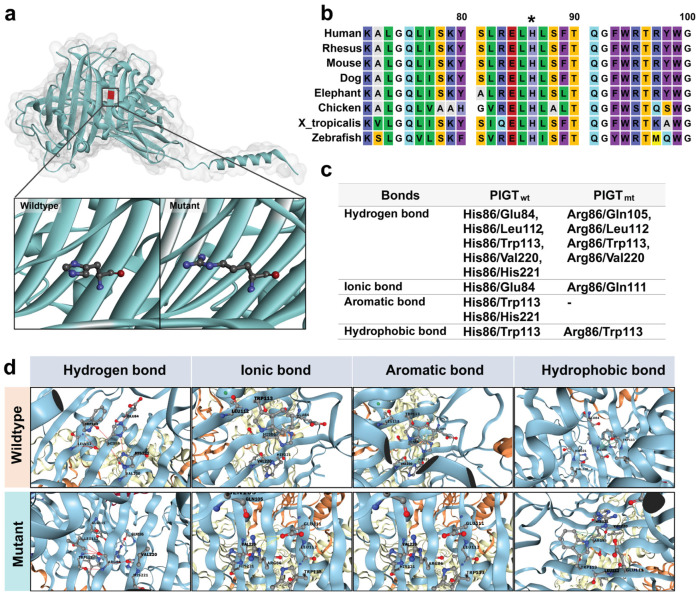
Prediction of protein stability at the alteration site through interaction formation. (**a**) Visualization performed using BIOVIA Discovery Studio Visualizer (Version 21.1.0.20298). (**b**) Conservation of amino acids around position 86 in PIGT across different species. An asterisk (*) indicates p.86 of the PIGT protein in each species. (**c**,**d**) Comparison of intramolecular bonds at the alteration site in wild-type (wt) and mutant (mt) proteins, computed and visualized via DDMut.

The three-dimensional structure of PIGT at the alteration site was predicted and compared between the wild-type and mutant. No significant change in protein folding resulting from the transition from His86 to Arg86 was found (Figure 4a). However, residue His86 is conserved across all species, and the amino acid sequence in this region of PIGT shows high conservation across species (Figure 4b). To assess alterations in intramolecular amino acid bonds within the mutational region, which could potentially impact the structure and function of the protein, we employed DDMut, a web-based machine learning tool (https://biosig.lab.uq.edu.au/ddmut/, accessed on 31 May 2024). DDMut uses sophisticated algorithms and data-driven predictions to provide insights into the structural consequences of the mutation [5]. In the wild-type PIGT protein, several intramolecular interactions were identified involving His86 and surrounding amino acids. These interactions included: (1) hydrogen bonds with Glu84, Leu112, Trp113, Val220, and His221; (2) an ionic bond with Glu84; (3) an aromatic bond with Trp113 and His221; and (4) hydrophobic interactions with Trp113. In contrast, in the PIGT mutant, Arg86 was predicted to interact with neighboring amino acids, including (1) hydrogen bonds with Gln105, Leu112, Trp113, and Val220; (2) an ionic bond with Gln111; (3) the absence of an aromatic bond; and (4) hydrophobic interaction with Trp113 (Figure 4c,d).

### 2.5. The Prediction of Protein Stability

The predictive analysis showed that the substitution of His with Arg at position 86 resulted in a ∆∆G value of −1.92 kcal/mol, indicating that the mutation led to protein destabilization.

## 3. Discussion

Neonatal hypotonia is a heterogeneous condition with several etiologies, including both congenital and acquired causes such as brain malformation, congenital infection, perinatal asphyxia, neonatal sepsis, and inborn errors of metabolism, including syndromic hypotonia [6]. Diagnosing this condition can be challenging, particularly in cases of syndromic monogenic hypotonia. This underscores the critical role of next-generation sequencing as a tool for achieving a definitive diagnosis of neonatal hypotonia.

In this study, we presented a newborn with hypotonia since birth who exhibited dysmorphic features. She also developed seizures and abnormal EEG findings, as previously reported in similar cases. Furthermore, the patient showed severe skeletal system manifestations, including a humeral fracture since birth. These clinical features were consistent with the spectrum of MCAHS3. Trio–WES analysis identified a novel homozygous missense variant of uncertain significance in the *PIGT* gene, known to be associated with MCAHS3. Our report broadens the spectrum of severe clinical manifestations observed in MCAHS3 and seeks to elucidate the significance of this novel variant in the *PIGT* gene. Additionally, this study represents the first report of this disease in the Thai population.

The c.257A>G variant in *PIGT* manifests as a severe phenotype of MCAHS3 in our patient. The reduced expression of CD59 in mononuclear cells supports impairment in GPI-AP synthesis in the patient. We investigated the amino acid residue change in the PIGT protein to elucidate the pathogenesis of this novel variant. We hypothesized that alterations in the tertiary structure of proteins due to amino acid modifications, even at a single locus, can affect protein folding kinetics, structural stability, dynamic flexibility, and overall molecular dynamics. According to computational predictions by DDMut, a negative ∆∆G value suggests that amino acid changes led to protein instability. This corresponds with observed alterations in bonds within the protein’s structure at the mutation site. Importantly, at the 86th amino acid position, the original His86 interacts significantly through hydrogen and ionic bonds with Glu84, a variant previously associated with reduced PIGT function in MCAHS3 syndrome [7]. While the PIGT (7wld) protein exhibits conservation in several amino acids, including Asp459, Tyr456, Asn461, Pro460, Phe522, Ser523, Asp521, Met524, and Asn527 [8], the GPI-binding site undergoing alteration lacks this conservation. Nevertheless, the region containing His86 is located within antiparallel β-strands and includes loops and short α-helices, which are crucial for facilitating interactions with GPAA1, PIGK, and PIGS—adjacent units essential for stabilizing the formation of the GPI transamidase complex [7]. Therefore, this mutation could potentially alter the attachment of the GPI anchor to the protein.

**Figure 5:**
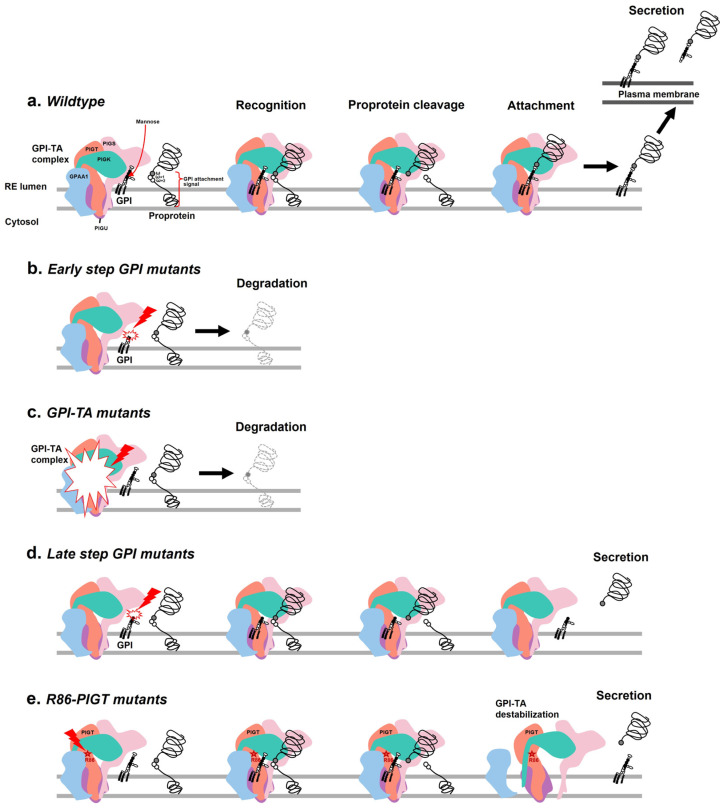
Models of different processing of GPI-AP synthesis in wild-type and defective cells with impaired GPI-AP synthesis. (**a**) In wild-type cells, the GPI-TA complex recognizes the mature GPI anchor and proprotein, leading to proprotein cleavage and attachment to the GPI anchor, resulting in normal cell surface expression and extracellular secretion. (**b**) In early-step mutations of GPI synthesis and (**c**) GPI-TA mutations, the GPI-TA complex does not recognize the mutant GPI anchor, resulting in proprotein degradation and a decrease in GPI-AP production and secretion. (**d**) Late-step GPI mutants contain mannose residues that are recognized by the GPI-TA complex, inducing proprotein cleavage but failing to attach to the mutant GPI, which leads to the secretion of the soluble form. (**e**) The GPI-TA complex containing the R86-PIGT mutant is predicted to be destabilized and not reach the attachment step, resulting in the secretion of cleaved proteins.

According to the process of GPI-AP synthesis, there are two main steps: GPI anchor synthesis and the attachment of the GPI anchor to the protein. The attachment process requires a specific and mature GPI anchor, a proprotein with a GPI attachment signal sequence containing a triplet of small amino acids (termed ω, ω + 1, and ω + 2), and the GPI-TA complex. Under wild-type conditions, after the GPI-TA complex recognizes the GPI anchor and the proprotein, GPI-TA cleaves the peptide bond between the ω and ω + 1 residues and attaches the GPI anchor to the ω site of the protein. For secreted proteins, the intact GPI-APs are then transported to the plasma membrane or released by specific mechanisms (Figure 5a). The impairment of GPI-AP synthesis due to either an ineffective GPI anchor backbone or GPI-TA complex can lead to two possible consequences. The first is intracellular degradation, exhibited by decreased surface expression. The second is the extracellular secretion of the protein in its inactive form without GPI anchor attachment [9].

ALP is a GPI-AP expressed in all tissues throughout the body and plays a key role in skeletal mineralization. Patients with MCAHS3 often exhibit low ALP levels (hypophosphatasia) along with abnormal skeletal features (Table 1). The latest study also found that patients with inherited glycosylphosphatidylinositol deficiency (IGD) disorders carrying *PIGT* mutations mostly exhibit low ALP levels (*n* = 4), while others have normal levels (*n* = 3) [10]. The low ALP level is particularly distinctive of a defect in the early step of GPI anchor biosynthesis or the loss of GPI-TA function [11]. Defects in enzymes involved in the early step of GPI synthesis result in premature GPI anchors that lack mannose, an important residue for GPI-TA recognition (Figure 5b). Consequently, the remaining proproteins are released from the GPI-TA complex and degraded within the cell. Similarly, in GPI-TA complex deficiency, GPI-AP synthesis is inhibited (Figure 5c). Both conditions reduce the expression of not only surface proteins but also secreted proteins. In contrast, our case is the first to report consistently high ALP levels (hyperphosphatasia) in a patient. Mayer et al. first reported a case with mental retardation and seizures, along with high ALP levels, associated with a defect in GPI-AP synthesis [12]. High ALP levels are a characteristic symptom of certain GPI deficiencies resulting from defects in enzymes involved in the late step of GPI anchor biosynthesis, such as PIGV, PIGO, PGAP2, PGAP3, and PIGW [13,14,15,16,17]. In this condition, premature GPI anchors still contain mannose, which can be recognized by GPI-TA and induce proprotein cleavage. However, the cleaved protein cannot be attached to the premature GPI anchor and is subsequently secreted (Figure 5d). In our case, the Arg86 variant of PIGT is predicted to induce protein destabilization, which might partially alter GPI-TA function. It is possible that this mutant GPI-TA is not stable enough to reach the attachment step, resulting in the secretion of cleaved proteins (Figure 5e). This hypothesis supports the clinical finding of elevated serum ALP levels and decreased CD59 expression in mononuclear cells derived from the patient.

## 4. Materials and Methods

### 4.1. Whole Exome Sequencing and Data Analysis

The genomic DNA was extracted from PBMC using the chloroform/isoamyl alcohol method. DNA quality and integrity were assessed using Nanodrop^®^ and 1.5% agarose gel electrophoresis. Subsequently, the DNA samples were sent to Macrogen^®^ (Seoul, Republic of Korea) for whole exome sequencing on an Illumina NovaSeq6000 platform, utilizing Agilent SureSelect V7 for exome capture with 12 G of data, producing 150-bp paired-end reads and achieving a coverage depth of 200×. The quality of the FASTQ files was checked using FASTQC software version 0.11.3 (https://www.bioinformatics.babraham.ac.uk/projects/fastqc/ accessed on 20 July 2023) [25], followed by adapter and primer removal from the raw read sequences using Cutadapt software version 3.1 [26]. The sequence of samples with at least 80% of raw read having a quality score (Phred score quality) > 35 were retained for further processing. The cleaned reads were then mapped to the human reference genome (hg19) using BWA-MEM software version 0.7.10 [27]. Variant calling files (VCF) were generated using HaplotypeCaller from GATK Best Practices (https://gatk.broadinstitute.org/hc/en-us, accessed on 20 July 2023) with default parameters.

### 4.2. Variant Analysis

The variant prioritization and interpretation were performed as a trio analysis. Variants were identified and evaluated using a custom collection of bioinformatic tools and comprehensively interpreted by our team of directors (MT and JK) and genetic counselors (MT and NM). Variants were filtered based on gene lists associated with the patient’s phenotype using HPO terms as of 17 October 2023, including HP:0001319 (neonatal hypotonia), HP:0001250 (seizure), and HP:0000939 (osteoporosis). The list of genes is provided in Table S1. Subsequently, variants with a minor allele frequency (MAF) greater than 0.05, based on data from the Genome Aggregation Database (gnomAD, https://gnomad.broadinstitute.org/, accessed on 17 October 2023) and the Thai Reference Exome database (T-REx, https://trex.nbt.or.th/, accessed on 17 October 2023), were excluded. The pathogenicity of missense variants was predicted using computational tools such as REVEL, BayesDel, M-CAP, and CADD. Variants classified as pathogenic (P), likely pathogenic (LP), and/or variants of uncertain significance (VUS) were comprehensively interpreted.

To confirm the variant existence, sequence data quality was assessed based on the standards and guidelines of the American College of Medical Genetics and Genomics (ACMG) and the Association for Molecular Pathology (AMP) 2015 [28]. The criteria included the following: (1) total read depth must be at least 20×, (2) alternative read depth should be 20–80% for heterozygous variants and 81–100% for homozygous variants, (3) genotype quality (GQX) must be greater than 20, (4) variant quality score must be greater than 30, and (5) the variant must be present in the BAM file in both forward and reverse strands. Candidate variant pathogenicity was classified according to ACMG/AMP 2015 guidelines, as well as additional guidelines from ClinGen (https://www.clinicalgenome.org/, accessed on 17 October 2023).

### 4.3. Sanger Sequencing

The variant validation and segregation were conducted using Sanger sequencing. Genomic DNA extracted from PBMC was used as the template for DNA amplification of exon 2 of the *PIGT* gene. Primers were designed using Primer-BLAST (https://www.ncbi.nlm.nih.gov/tools/primer-blast/, accessed on 22 January 2024). The primer sequences are as follows: *PIGT*-F: 5′-GCGGGAGGAACTTGTCATCA-3′ and *PIGT*-R: 5′-TGCCTACTGGAAGTGCTGTC-3′. PCR reactions were performed using Phusion^TM^ high-fidelity DNA polymerase (Thermo Scientific, Waltham, MA, USA). The 552 bp PCR product was sequenced by ATGC Co., Ltd. (WardMedic, Thailand) using the pair of exon 2 *PIGT* primers.

### 4.4. Western Blot Analysis for CD59 Expression

Given the function of PIGT, the potential effects of the mutation on GPI-anchored proteins (GPI-APs) were investigated. PBMCs were the only available biological sample from the patient; therefore, CD59 expression on mononuclear cells was chosen as the representative GPI-AP to assess GPI-TA function, as supported by previous studies [4,7,22]. Although a broader analysis of GPI-APs (e.g., CD16, CD14, CD48, CD24, or FLAER staining) would have been ideal, fresh blood samples were not available due to the patient’s passing. Since the PBMCs were preserved as cell pellets at −80 °C, the sample was not suitable for flow cytometry, and thus, western blot analysis was performed as the most feasible approach. PBMC pellets from the patient, parents, and age-matched wild-type *PIGT* controls were used for the experiment. PBMC pellets were lysed in ice-cold RIPA buffer (50 mM Tris-HCl pH 8.0, 150 mM NaCl, 1% NP-40, 0.5% sodium deoxycholate, 0.1% SDS, 1 mM EDTA) containing protease and phosphatase inhibitors. Protein concentration was determined using the BCA protein assay kit (Pierce^TM^, Waltham, MA, USA). Fifteen micrograms of protein were separated into 12.5% SDS-polyacrylamide gels and transferred to nitrocellulose membranes. The membranes were blocked in a solution of 10% skim milk in TBST at room temperature for 1 h, followed by incubation with rabbit anti-human CD59 antibody (1:1000; ab133707, Abcam, Cambridge, UK) or rabbit anti-human β-actin antibody (1:3000; ab8227, Abcam, Cambridge, UK) in TBST at 4 °C overnight. Subsequently, the membranes were washed and incubated with horseradish peroxidase-conjugated anti-rabbit antibody for 1 h at room temperature. Bands were detected using SuperSignal^TM^ West Femto substrate (Thermo Scientific, Waltham, MA, USA) and visualized using the ChemiDoc^TM^ Imaging System (BIO-RAD, Hercules, CA, USA).

### 4.5. In-Silico Analysis of the PIGT Mutation on Protein Stability and Structural Dynamics

We hypothesized that the p.His86Arg mutation in PIGT may contribute to the onset of MCAHS3-related diseases, primarily due to its impact on protein folding, thermodynamic stability, and associated interactions. Therefore, we utilized in silico biophysical methodologies, specifically DDMut (https://biosig.lab.uq.edu.au/ddmut, accessed on 31 May 2024) [5], to predict the subtle structural changes induced by this mutation. DDMut is a valuable tool for forecasting alterations in protein stability, quantified in terms of ∆∆G (in kcal/mol). A positive ∆∆G value (∆∆G > 0 kcal/mol) indicates protein stabilization, while a negative ∆∆G value (∆∆G < 0 kcal/mol) suggests protein destabilization. The three-dimensional structures of both the wild-type and mutant PIGT (PDB: 7wld) were visualized using BIOVIA software [29].

## 5. Conclusions

Summarizing the results from the in-silico predictions, including the observed structural alterations and significant changes in intramolecular interactions at position 86 of PIGT, suggests that the p.His86Arg mutation may impact GPI-TA complex activity. However, further functional analysis is needed to understand the relevance of this mutation in PIGT function and its effect on patient phenotypes within the context of MCAHS3 syndrome.

## Figures and Tables

**Table 1:** Clinical features of patients diagnosed with MCAHS3 syndrome due to *PIGT* variants.

Study	Our Study	Ranjan A. 2023 [18]	Hur Y. J. 2021 [19]	Kohashi K. 2018 [20]	Yang L. 2018 [21]	Pagnamenta A.T. 2017 [22]	Skauli N. 2016 [23]	Lam C. 2015 [24]	Nakashima M. 2014 [7]	Kvarnung M. 2013 [4]
Case number	1	1	1	1	1	3	2	2	1	4
Sex	Female	Male	Female	Male	Male	1 Female/ 2 Male	Male	1 Male 1 Female	Female	Female
Consanguinity	Yes	No	No	No	No	NA	Yes	No	No	Yes
Ethnicity	Thai	Indian	Korean	Japanese	Chinese	Caucasian/ Afghanistan	Somalian	NA	Japanese	Turkish
Dysmorphic features	+	+	+	+	+	+/NA	+	+	NA	+
Hypotonia	+	+	+	+	+	NA	+	+	+	+
Seizure	+	+	+	+	+	+	+	+	+	+
Seizure type	Myoclonic jerk	GTC, myoclonic, tonic	GTC	Myoclonic, Tonic, GTC, epileptic apnea	Myoclonic	GTC	Myoclonic, Tonic, GTC, complex partial seizures	Tonic, myoclonic	Myoclonic, Tonic, GTC	Myoclonic, GTC, head jerk, blinking, Absence
Brain atrophy	+	NA	+	+	+	+	+	+	+	+(3/4)
EEG	Multifocal epileptiform discharges from the right central parietal, left temporal, and left frontal areas	NA	Normal background rhythm, no epileptiform discharges	High-amplitude slow wave	Slow background wave	bilateral slow activity intermixed with sharp and spike waves /NA	Multiple spike-wave	Multifocal epileptiform, theta waves	High-amplitude slow wave	Multifocal epileptiform, theta waves
AED	LEV, PB	PB, FOS, LEV, CZP	VPA	LEV	LEV	NA/NA	NA	LEV, TPM, PB, CZP, MZ, BDZ, CLN, and ketogenic diet	CBZ, CLO, PLP, VPA, ZNS, PHE	NA
Developmental delay	Severe	NA	Severe	Severe	Severe	Severe/ Profound	Severe/ Moderate-severe	Profound	Profound	Severe
Skeletal	+	+	Normal	+	Normal	+/NA	Normal	+	+	+
Alkaline phosphatase level	High	Low	Low	Low	Normal	Normal/ Low, Normal	Normal	Normal	Low	Low
Variants in *PIGT*	Homozygous c.257A>G (p.His86Arg)	Homozygous c.709G>C (p.Glu237Gln)	c.250G>T (p.Glu84*) and c.1582G>A (p.Val528Met)	c.250 G>T (p.Glu84*) and c.1096 G>T (p.Gly366Trp)	Homozygous c.550G>A (p.Glu184Lys)	c.1582G4A (p.Val528Met) and c.1730dupC (p.Leu578fs*35) Homozygous c.709G>C (p.Glu237Gln)	Homozygous c.1079G>T (p.Gly360Val)	c.918dupC (p.Val307Argfs*13) and c.1342C>T (p.Arg448Trp)	c.250G>T (p.Glu84*) and c.1342C>T (p.Arg488Trp)	Homozygous c.547A>C (p.Thr183Pro)

BDZ: benzodiazepine; CBZ: carbamazepine; CLN: clonidine; CLO: clobazam; CZP: clonazepam; FOS: fosphenytoin; GTC: generalized tonic-clonic seizures; LEV: levetiracetam; MZ: midazolam; PB: phenobarbital; PHE: phenytoin; PLP: pyridoxal phosphate; TPM: topiramate; VPA: valproic acid; ZNS: zonisamide; +: affected.

## Data Availability

The authors confirm that the data supporting the findings of this study are available within the article.

## Supplementary Materials

The following supporting information can be downloaded at https://www.mdpi.com/article/10.3390/ijms26062790/s1.

## Abbreviations

The following abbreviations are used in this manuscript:

PIGT	Phosphatidylinositol glycan class T
GPI-TA	Glycosylphosphatidylinositol transamidase
GPI-AP	Glycosylphosphatidylinositol-anchored protein
MCAHS3	Multiple Congenital Anomalies-Hypotonia Seizures Syndrome 3
WES	Whole exome sequencing
ALP	Alkaline phosphatase
ER	Endoplasmic reticulum
EEG	Electroencephalogram
ACMG/AMP	The American College of Medical Genetics and Genomics and the Association for Molecular Pathology
PBMC	Peripheral blood mononuclear cell
VCF	Variant calling file
HPO	Human phenotype ontology
GnomAD	The Genome Aggregation Database
T-REx	The Thai Reference Exome Database
