# Supplementary material for: A Novel Homozygous Missense Variant of PIGT Related to Multiple Congenital Anomalies-Hypotonia Seizures Syndrome 3 with Elevated of Serum ALP Level in a Thai Newborn Patient
Source: Int J Mol Sci. 2025 Mar 20;26(6):2790. doi: 10.3390/ijms26062790 (PMC11943126; doi:10.3390/ijms26062790)

Figure S1. The BAM captures demonstrate the existence of the *PIGT*: c.257A>G (p.His86Arg) in the patient in a homozygous state (SP046) and in her parent with a heterozygous state (SP047: father and SP048: mother).

Proband

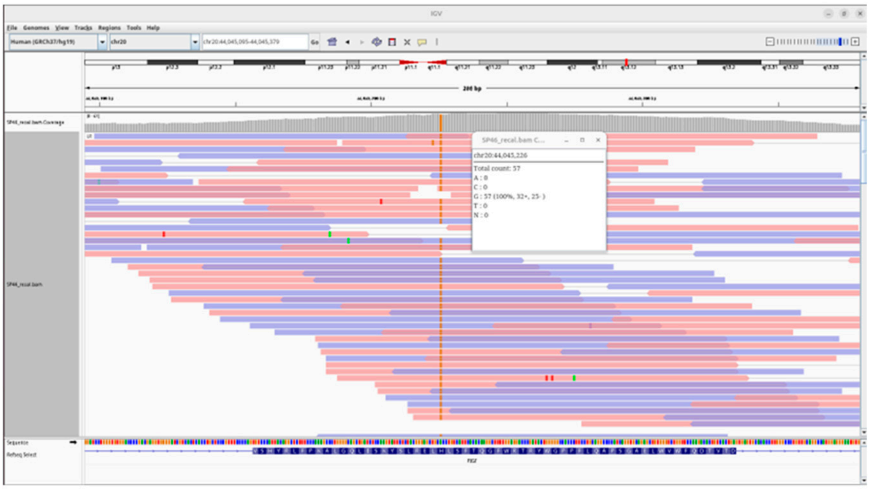

Father

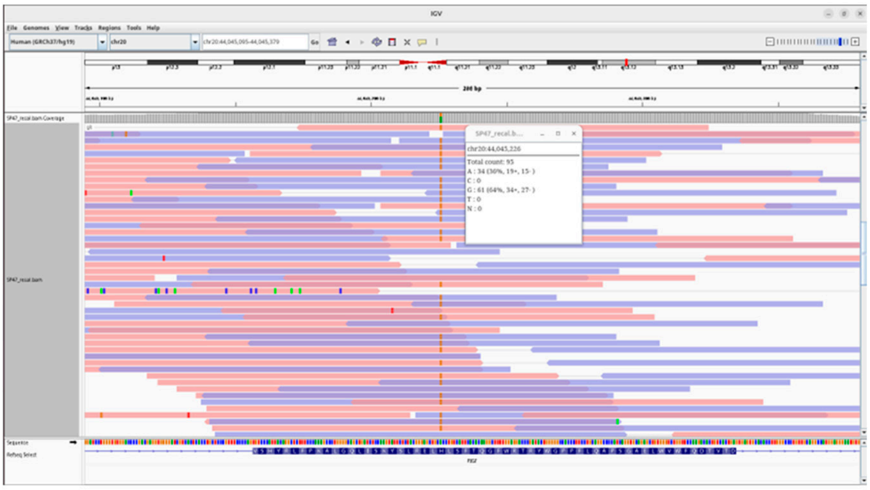

Mother

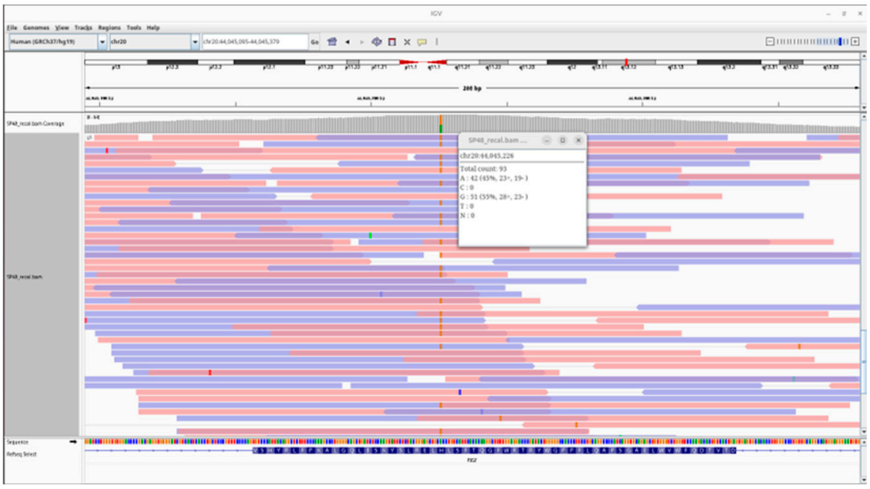

Supplement: Supplementary file 1 [file ijms-26-02790-s001.zip › Figure S1.pdf]
