# Supplementary material for: A Novel Homozygous Missense Variant of PIGT Related to Multiple Congenital Anomalies-Hypotonia Seizures Syndrome 3 with Elevated of Serum ALP Level in a Thai Newborn Patient
Source: Int J Mol Sci. 2025 Mar 20;26(6):2790. doi: 10.3390/ijms26062790 (PMC11943126; doi:10.3390/ijms26062790)
Supplement: Supplementary file 1 [file ijms-26-02790-s001.zip › Table S1.pdf]

Table S1. Gene list associated with patient phenotypes used in the variant filtering.

**HPO term**	**Gene list**
HP:0001319, Neonatal hypotonia	*ACOX1, ACTA1, ADNP, AFG2A, ALG11, AP4B1, AP4E1, AP4M1, ARID1B, ARX, ATP2B3, ATXN7, B3GALT6, BCS1L, BIN1, BPTF, BRAF, CAMTA1, CASK, CCDC174, CCDC88A, CDKL5, CFL2, CHRNA1, CHRNB1, CHRND, CHRNE, CLPB, CNTN1, COA6, COG4, COL12A1, COL6A1, COPB1, CPT2, CRPPA, CWC27, DLAT, DLK1, DMPK, DNAJC19, DST, EARS2, EEF1A2, EGR2, EIF5A, ERCC6L2, FGFR1, FIG4, FKBP14, FKRP, FKTN, FLNA, FOXG1, FXR1, GABBR2, GABRA2, GATA6, GATAD2B, GDAP1, GLYCTK, GNPTAB, GOT2, GRIA4, H1-4, HADH, HERC2, HNF1A, HNF4A, HSD17B4, IER3IP1, IGHMBP2, IPW, ITPR1, KANSL1, KAT6A, KCNK9, KLHL41, KNSTRN, LAMA2, LARGE1, LMOD3, LYRM4, MADD, MAG, MAGEL2, MECP2, MED12, MEG3, MEGF10, MKRN3, MRPS16, MT-TE, MT-TT, MTHFR, MTM1, MUSK, MYT1L, NADK2, NALCN, NDN, NEB, NEXMIF, NFIX, NONO, NPAP1, NR4A2, NSD1, NTNG1, OCRL, ODC1, PAFAH1B1, PCLO, PDE4D, PDHX, PDP1, PDSS2, PEX1, PEX10, PEX12, PEX16, PEX2, PEX26, PEX3, PEX6, PGAP1, PHIP, PIEZO2, PIK3CD, PLCB3, PLOD1, POMK, POMT1, POMT2, POU4F1, PPP2R5D, PRDM5, PRKAR1B, PRPS1, PSMD12, PTRH2, PURA, PWAR1, PWRN1, PYROXD1, RAPSN, RBL2, RPL10, RTL1, RYR1, SCN4A, SDHA, SH2B1, SHANK3, SIM1, SLC12A6, SLC16A2, SLC25A22, SLC25A46, SLC6A8, SLF2, SMC1A, SNORD115-1, SNORD116-1, SNRPN, SON, SOX10, SPOP, SPTBN4, STAG1, SUCLG1, SUMF1, SYNE1, SYT1, TAF6, TBC1D24, TBCK, TBR1, TGFB3, TIMM22, TMCO1, TMEM260, TNNT1, TPM2, TPM3, TSFM, TUBA8, TUFM, UNC80, UPB1, UQCC2, USP7, VAC14, VMA21, VPS13B, VPS53, WAC, WDR19, ZNF148, ZNF469*
HP:0001250, Seizure	*AAAS, AARS1, AASS, ABAT, ABCA2, ABCA5, ABCA7, ABCB7, ABCC6, ABCC8, ABCD1, ABHD16A, ACADM, ACADS, ACADSB, ACADVL, ACAT1, ACBD6, ACO2, ACOX1, ACP5, ACSF3, ACTA2, ACTB, ACTG1, ACTL6B, ACVR1, ACVRL1, ACY1, ADA2, ADAM22, ADAMTS3, ADAMTSL2, ADAR, ADARB1, ADAT3, ADD3, ADGRG1, ADGRL1, ADGRV1, ADK, ADNP, ADORA2A, ADPRS, ADRA2B, ADSL, AFF3, AFG2A, AFG2B, AFG3L2, AGA, AGO1, AGO2, AGRN, AGTPBP1, AHDC1, AHI1, AHSG, AIFM1, AIMP1, AIMP2, AIP, AIPL1, AIRE, AKAP9, AKT1, AKT2, AKT3, ALDH18A1, ALDH3A2, ALDH4A1, ALDH5A1, ALDH7A1, ALDOB, ALG1, ALG11, ALG12, ALG13, ALG14, ALG2, ALG3, ALG6, ALG8, ALG9, ALK, ALKBH8, ALMS1, ALPL, ALX4, AMACR, AMER1, AMFR, AMPD2, AMT, ANGPTL6, ANK1, ANK2, ANK3, ANKH, ANKLE2, ANKRD11, ANKRD17, ANO10, ANOS1, ANTXR1, AP1G1, AP1S2, AP2M1, AP3B2, AP3D1, AP4B1, AP4E1, AP4M1, AP4S1, APC2, APOE, APP, AQP2, AQP4, ARCN1, ARF1, ARFGEF1, ARFGEF2, ARG1, ARHGAP31, ARHGDIA, ARHGEF9, ARID1A, ARID1B, ARID2, ARL13B, ARL3, ARL6, ARL6IP6, ARMC9, ARNT2, ARSA, ARSB, ARV1, ARVCF, ARX, ASAH1, ASCC3, ASCL1, ASH1L, ASL, ASNS, ASPA, ASPM, ASS1, ASXL1, ASXL2, ASXL3, ATAD1, ATAD3A, ATG7, ATIC, ATM, ATN1, ATP10A, ATP11A, ATP13A2, ATP1A1, ATP1A2, ATP1A3, ATP2A2, ATP2B1, ATP5F1A, ATP5F1D, ATP5F1E, ATP5MK, ATP5PO, ATP6AP1, ATP6AP2, ATP6V0A1, ATP6V0A2, ATP6V0C, ATP6V1A, ATP6V1B2,*

	ATP6V1E1, ATP7A, ATP7B, ATP8A2, ATP9A, ATPAF2, ATR, ATRX, ATXN10, AUH, AUTS2, AVPR2, B3GALNT2, B3GALT6, B3GLCT, B4GAT1, B9D1, B9D2, BANK1, BAP1, BBIP1, BBS1, BBS10, BBS12, BBS2, BBS4, BBS5, BBS7, BBS9, BCAP31, BCAS3, BCKDHA, BCKDHB, BCKDK, BCL10, BCL11B, BCOR, BCORL1, BCR, BCS1L, BDNF, BICRA, BLK, BLTP1, BMP2, BMP4, BMPR1A, BOLA3, BPTF, BRAF, BRAT1, BRCA2, BRD4, BRPF1, BSCL2, BTD, BUB1, BUB1B, BUB3, C12orf57, C1GALT1C1, C2orf69, C4A, C4B, CA8, CABP4, CACNA1A, CACNA1B, CACNA1C, CACNA1D, CACNA1E, CACNA1G, CACNA1H, CACNA1I, CACNA2D1, CACNA2D2, CACNB4, CACNG2, CAD, CALM1, CALM2, CALM3, CAMK2A, CAMK2B, CAMK2G, CAMKMT, CAMLG, CAMSAP1, CAMTA1, CAPRIN1, CARS1, CARS2, CASK, CASP10, CASP2, CASQ2, CASR, CASZ1, CAV3, CBS, CBY1, CC2D2A, CCBE1, CCDC115, CCDC141, CCDC47, CCDC78, CCDC88A, CCDC88C, CCM2, CCND2, CCNQ, CCR1, CD96, CDC40, CDC42, CDH11, CDH15, CDH2, CDH23, CDK10, CDK13, CDK19, CDK5, CDK6, CDK8, CDKL5, CDKN1A, CDKN1B, CDKN2B, CDKN2C, CDON, CELF2, CENPE, CEP104, CEP120, CEP152, CEP164, CEP19, CEP290, CEP41, CEP57, CEP85L, CERS1, CERT1, CFAP418, CFH, CFHR1, CFHR3, CHAT, CHD1, CHD2, CHD3, CHD5, CHD7, CHD8, CHEK2, CHKB, CHMP2B, CHN1, CHRNA2, CHRNA4, CHRNA7, CHRNB2, CIC, CILK1, CISD2, CIT, CKAP2L, CLCF1, CLCN2, CLCN3, CLCN4, CLCN6, CLCNKB, CLDN16, CLEC7A, CLIC2, CLN3, CLN5, CLN6, CLN8, CLP1, CLPB, CLPP, CLTC, CLTCL1, CLTRN, CMPK2, CNKSR2, CNNM2, CNOT1, CNPY3, CNTN2, CNTNAP1, CNTNAP2, COA8, COG2, COG3, COG4, COG5, COG6, COG7, COG8, COL13A1, COL18A1, COL1A2, COL3A1, COL4A1, COL4A2, COLGALT1, COMT, COPB1, COPB2, COQ2, COQ4, COQ5, COQ6, COQ8A, COQ9, COX10, COX11, COX4I1, COX7B, COX8A, CPA6, CPAP, CPLANE1, CPLX1, CPOX, CPS1, CPSF3, CPT1A, CPT1C, CPT2, CR2, CRADD, CRB1, CRB2, CRBN, CREBBP, CRELD1, CRH, CRIPT, CRIPTO, CRKL, CRLF1, CRLS1, CRPPA, CRX, CSF1R, CSNK2A1, CSNK2B, CSPP1, CSTB, CTBP1, CTC1, CTCF, CTH, CTLA4, CTNNA2, CTNNB1, CTNND2, CTNS, CTSA, CTSD, CTSF, CTU2, CUL3, CUL4B, CUX2, CXCR4, CYB5A, CYB5R3, CYFIP2, CYP26C1, CYP27A1, CYP27B1, CYP2R1, D2HGDH, DAG1, DALRD3, DARS1, DARS2, DBH, DBR1, DCC, DCPS, DCX, DDB2, DDC, DDOST, DDX3X, DDX59, DEAF1, DEGS1, DENND5A, DEPDC5, DGUOK, DHCR24, DHCR7, DHDDS, DHFR, DHPS, DHX16, DHX30, DHX37, DHX9, DIAPH1, DIP2B, DIS3L2, DISP1, DLD, DLG3, DLG4, DLK1, DLL1, DLL4, DMP1, DMXL2, DNAJC19, DNAJC5, DNAJC6, DNASE1, DNASE1L3, DNM1, DNM1L, DNMT3A, DNMT3B, DOCK6, DOCK7, DOHH, DOLK, DPAGT1, DPF2, DPH1, DPH2, DPH5, DPM1, DPM2, DPM3, DPP9, DPYD, DPYS, DPYSL5, DRG1, DSTYK, DTYMK, DUSP6, DUX4, DUX4L1, DVL1, DYNC1H1, DYNC1I2, DYRK1A, EARS2, EBF3, EBP, ECE1, ECM1, EDN3, EED, EEF1A2, EEF2, EFHC1, EFTUD2, EGF, EHMT1, EIF2AK2, EIF2AK3, EIF2B1, EIF2B3, EIF2S3, EIF3F, EIF4A2, ELMO2, ELOVL4, ELP1, EMC1, EMC10, EMG1, EML1, EN1, ENG, ENPP1, EOGT, EP300, EPCAM, EPG5, EPHB4, EPM2A, EPRS1, ERAP1, ERCC1, ERCC2, ERCC3, ERCC4, ERCC5, ERCC6, ERCC8, ERLIN2, ERMARD, ESAM, ETHE1, ETS1, EXOC2, EXOC7,

	EXOC8, EXOSC3, EXOSC5, EXOSC8, EXOSC9, EXT2, EXTL3, EZH2, F8, FA2H, FADD, FAM111A, FAM149B1, FAM50A, FAR1, FARS2, FARSB, FAS, FASLG, FASTKD2, FAT4, FBLN1, FBP1, FBP2, FBXL4, FBXO11, FBXO28, FBXW7, FCGR2A, FCGR2B, FCGR3B, FCSK, FDFT1, FDXR, FEZF1, FGF10, FGF12, FGF13, FGF17, FGF8, FGFR1, FGFR2, FGFR3, FGFRL1, FH, FIG4, FILIP1, FITM2, FKRP, FKTN, FLCN, FLI1, FLII, FLNA, FLRT3, FLVCR1, FLVCR2, FMN2, FMR1, FOLR1, FOSL2, FOXA2, FOXG1, FOXH1, FOXP1, FOXP3, FOXRED1, FRA10AC1, FRA16E, FRAS1, FRG1, FRMD5, FRMPD4, FRRS1L, FTH1, FTL, FTO, FTSJ1, FUCA1, FUT8, FXYD2, FZR1, GABBR1, GABBR2, GABRA1, GABRA2, GABRA3, GABRA5, GABRB1, GABRB2, GABRB3, GABRD, GABRG2, GAD1, GAL, GALC, GALK1, GALNT2, GALT, GAMT, GAS1, GATA3, GATA4, GATA6, GATAD2B, GBA1, GCDH, GCH1, GCK, GCM2, GCSH, GDF2, GDF6, GDI1, GDNF, GEMIN4, GET4, GFAP, GFM1, GFM2, GJA1, GJA5, GJA8, GJC2, GK, GLA, GLB1, GLDC, GLE1, GLI2, GLI3, GLRA1, GLRA2, GLRB, GLRX5, GLS, GLUD1, GLUL, GLYCTK, GM2A, GMPPA, GMPPB, GNA11, GNAI1, GNAO1, GNAQ, GNAS, GNB1, GNB2, GNB5, GNE, GNS, GOLGA2, GON7, GOSR2, GOT2, GP1BA, GP1BB, GP9, GPAA1, GPC3, GPC4, GPHN, GPR101, GPR161, GPRC5B, GPSM2, GPT2, GRIA1, GRIA2, GRIA3, GRIA4, GRIK2, GRIN1, GRIN2A, GRIN2B, GRIN2D, GRM1, GRM7, GRN, GSS, GTF2E2, GTF2H5, GTPBP2, GTPBP3, GUCY1A1, GUCY2D, GUF1, GYS1, GYS2, H19, H3-3A, H4C3, H4C5, HACE1, HADH, HADHA, HADHB, HAX1, HCCS, HCFC1, HCN1, HCN2, HCN4, HDAC4, HDAC8, HECTD4, HECW2, HEPACAM, HERC1, HERC2, HESX1, HEXA, HEXB, HGSNAT, HHAT, HIBCH, HIC1, HID1, HIRA, HIVEP2, HK1, HLA-B, HLA-DPA1, HLA-DPB1, HLA-DQA1, HLA-DQB1, HLA-DRB1, HLCS, HMBS, HMGCL, HMGCS2, HNF1A, HNF1B, HNF4A, HNRNPC, HNRNPH1, HNRNPH2, HNRNPK, HNRNPR, HNRNPU, HOXA1, HPD, HPDL, HPRT1, HRAS, HS6ST1, HS6ST2, HSD17B10, HSD17B4, HSPD1, HSPG2, HTRA1, HTRA2, HTT, HUWE1, HYCC1, HYLS1, HYMAI, IARS1, IBA57, IDH2, IDS, IER3IP1, IFIH1, IFNG, IFNGR1, IFT140, IFT172, IFT27, IFT56, IFT74, IGF2, IGHG1, IKBKG, IL10, IL11RA, IL12A, IL12A-AS1, IL12B, IL17F, IL17RA, IL17RC, IL17RD, IL1RAPL1, IL23R, IMPDH1, INPP5E, INPP5K, INS, INSR, INTS1, INTS11, INTS8, IPW, IQCB1, IQSEC1, IQSEC2, IRAK1, IREB2, IRF2BPL, IRF3, IRF4, IRF5, ISCA1, ISG15, ITGAM, ITGB6, ITPA, ITPR1, IVD, JAK2, JAM2, JAM3, JARID2, JAZF1, JMJD1C, JRK, KANSL1, KARS1, KAT5, KAT6A, KAT6B, KAT8, KATNB1, KATNIP, KCNA1, KCNA2, KCNAB2, KCNB1, KCNC1, KCNC2, KCNC3, KCNE1, KCNE2, KCNH1, KCNH2, KCNH5, KCNJ1, KCNJ10, KCNJ11, KCNJ13, KCNJ16, KCNJ2, KCNJ5, KCNJ6, KCNK4, KCNMA1, KCNN2, KCNN3, KCNQ1, KCNQ2, KCNQ3, KCNQ5, KCNT1, KCNT2, KCTD7, KDM3B, KDM4B, KDM5A, KDM5C, KDM6A, KDM6B, KIAA0319L, KIAA0586, KIAA0753, KIF11, KIF15, KIF1A, KIF22, KIF26A, KIF2A, KIF4A, KIF5A, KIF5C, KIF7, KIFBP, KLF13, KLHL15, KLLN, KLRC4, KMT2A, KMT2C, KMT2D, KMT2E, KMT5B, KNL1, KNSTRN, KPNA3, KPTN, KRAS, KRIT1, L1CAM, L2HGDH, LAGE3, LAMA2, LAMA3, LAMB1, LAMB3, LAMC2, LAMC3, LARGE1, LARP7, LARS1, LARS2, LAS1L, LBR, LBX1, LCA5, LETM1, LGI1, LGI4, LHX1, LHX4, LIAS, LIFR, LIG4, LINGO1, LIPT2,

	LMAN2L, LMBRD1, LMBRD2, LMNB1, LMNB2, LMX1B, LNPK, LONP1, LRAT, LRP2, LRP5, LRPPRC, LRRK1, LSM11, LSS, LUZP1, LYST, LZTFL1, LZTR1, MACF1, MADD, MAF, MAFB, MAGEL2, MAN1B1, MANBA, MAP1B, MAP2K1, MAPK1, MAPK10, MAPK8IP3, MAPRE2, MAPT, MARCHF6, MAST1, MAST3, MBD5, MBOAT7, MBTPS2, MC2R, MCCC1, MCCC2, MCPH1, MCTP2, MDH1, MDH2, MECP2, MED11, MED12, MED12L, MED13, MED17, MED25, MED27, MEF2C, MEFV, MEG3, MEGF10, MEGF8, MEIS2, MEN1, METTL23, METTL5, MFF, MFSD2A, MFSD8, MGAT2, MGP, MICAL1, MICOS13, MICU1, MID2, MINPP1, MIPEP, MKKS, MKRN3, MKS1, MLC1, MLH1, MLX, MLYCD, MMAA, MMACHC, MMADHC, MMP23B, MMUT, MN1, MOCS1, MOCS2, MOGS, MORC2, MPC1, MPDU1, MPDZ, MPL, MPLKIP, MPV17, MRAP, MRM2, MRPL12, MRPS22, MRPS34, MSH2, MSH6, MSL3, MSX2, MT-ATP6, MT-ATP8, MT-CO1, MT-CO2, MT-CO3, MT-CYB, MT-ND1, MT-ND2, MT-ND3, MT-ND4, MT-ND5, MT-ND6, MT-RNR1, MT-TC, MT-TF, MT-TH, MT-TI, MT-TK, MT-TL1, MT-TN, MT-TP, MT-TQ, MT-TS1, MT-TS2, MT-TT, MT-TV, MT-TW, MTFMT, MTHFD1, MTHFR, MTHFS, MTO1, MTOR, MTR, MTRR, MTSS2, MUTYH, MVK, MYCN, MYH14, MYH3, MYMK, MYO1H, MYO5A, MYO9A, MYORG, MYRF, MYT1L, NAA10, NAA15, NAA60, NACC1, NADK2, NAE1, NAGA, NAGLU, NAGS, NALCN, NANS, NAPB, NARS1, NARS2, NAT8L, NAXD, NAXE, NBAS, NBEA, NCAPD3, NCDN, NDE1, NDN, NDNF, NDP, NDST1, NDUFA1, NDUFA11, NDUFA13, NDUFA2, NDUFA4, NDUFA6, NDUFA8, NDUFAF2, NDUFAF3, NDUFAF4, NDUFAF5, NDUFAF6, NDUFAF8, NDUFB11, NDUFB8, NDUFC2, NDUFS1, NDUFS4, NDUFS6, NDUFS7, NDUFS8, NDUFV1, NECAP1, NECTIN1, NEDD4L, NEK1, NEK9, NELFA, NEU1, NEUROD2, NEUROG1, NEXMIF, NF1, NF2, NFASC, NFIA, NFIB, NFIX, NFKB2, NFS1, NFU1, NGLY1, NHLRC1, NHLRC2, NIN, NIPA1, NIPA2, NIPBL, NKX6-2, NLGN3, NLGN4X, NLRP3, NMNAT1, NNT, NODAL, NONO, NOS1AP, NOS3, NOTCH1, NOTCH2NLC, NOTCH3, NOVA2, NPAP1, NPC1, NPC2, NPHP1, NPRL2, NPRL3, NR0B1, NR2F1, NR4A2, NRAS, NRROS, NSD1, NSD2, NSDHL, NSF, NSRP1, NSUN2, NSUN3, NSUN6, NTNG1, NTNG2, NTRK2, NUBPL, NUP107, NUP133, NUP214, NUS1, OAT, OCA2, OCLN, OCRL, ODC1, OFD1, OGDH, OGDHL, OPA1, OPHN1, OSGEP, OSTM1, OTC, OTUD5, OTUD6B, OTUD7A, OTX2, OXR1, P4HTM, PACS1, PACS2, PAFAH1B1, PAH, PAK1, PAK2, PAK3, PANK2, PARS2, PAX2, PC, PCCA, PCCB, PCDH12, PCDH19, PCDHGC4, PCK1, PCLO, PCNT, PCSK1, PCYT1A, PCYT2, PDCD1, PDCD10, PDCD6IP, PDE10A, PDE2A, PDE4D, PDE6D, PDGFB, PDGFRB, PDHA1, PDHB, PDHX, PDP1, PDPN, PDSS2, PDX1, PDZD8, PEPD, PERP, PET100, PEX1, PEX10, PEX11B, PEX12, PEX13, PEX14, PEX16, PEX19, PEX2, PEX26, PEX3, PEX5, PEX6, PEX7, PGAP1, PGAP2, PGAP3, PGK1, PGM2L1, PGM3, PHACTR1, PHF21A, PHF6, PHGDH, PHIP, PHOX2B, PI4K2A, PI4KA, PIBF1, PIDD1, PIEZO2, PIGA, PIGB, PIGC, PIGF, PIGG, PIGH, PIGK, PIGL, PIGM, PIGN, PIGO, PIGP, PIGQ, PIGS, PIGT, PIGU, PIGV, PIGW, PIGY, PIK3CA, PIK3CD, PIK3R2, PLA2G6, PLAA, PLAGL1, PLCB1, PLCH1, PLEKHG2, PLK4, PLP1, PLPBP, PLXNA1, PMM2, PMPCB, PMS1, PMS2, PNKP, PNPLA6, PNPLA8,

	PNPO, PODXL, POGZ, POLA1, POLD1, POLE, POLG, POLG2, POLR1A, POLR2A, POLR3A, POLR3B, POLRMT, POMC, POMGNT1, POMGNT2, POMK, POMP, POMT1, POMT2, POU1F1, POU3F3, POU3F4, POU4F1, PPA2, PPFIBP1, PPIL1, PPM1B, PPOX, PPP1R15B, PPP1R21, PPP2CA, PPP2R1A, PPP2R5D, PPP3CA, PPT1, PQBP1, PRDM13, PRDM16, PRDM8, PRDX1, PREPL, PRF1, PRICKLE1, PRKAG2, PRKAR1B, PRKCZ, PRKDC, PRMT7, PRNP, PROC, PRODH, PROK2, PROKR2, PROP1, PRORP, PROS1, PRPS1, PRRT2, PRTN3, PRUNE1, PSAP, PSAT1, PSEN1, PSEN2, PSMB8, PSMB9, PSMD12, PSPH, PTCH1, PTEN, PTF1A, PTH, PTH1R, PTPN22, PTPN23, PTRH2, PTRHD1, PTS, PUF60, PUM1, PURA, PUS3, PWAR1, PWRN1, PXK, PYCR2, QARS1, QDPR, RAB11B, RAB18, RAB27A, RAB39B, RAB3GAP1, RAB3GAP2, RAC1, RAC3, RAD21, RAF1, RAI1, RALA, RALGAPA1, RANBP2, RAP1GDS1, RAPGEF2, RARS1, RARS2, RASA1, RBCK1, RBL2, RBM10, RBM8A, RBMX, RBPJ, RD3, RDH12, RECQL4, RELN, RERE, RET, RFT1, RFX5, RFX7, RHOBTB2, RMND1, RNASEH2A, RNASEH2B, RNASEH2C, RNASET2, RNF113A, RNF125, RNF13, RNF2, RNF213, RNF220, RNH1, RNU12, RNU4-2, RNU4ATAC, RNU7-1, ROBO1, ROBO3, ROGDI, RORA, RORB, RPE65, RPGRIP1, RPGRIP1L, RPIA, RPL10, RPS20, RPS6KA3, RRAGD, RREB1, RRM2B, RSRC1, RTL1, RTN2, RTN4IP1, RTTN, RUBCN, RUSC2, RXYLT1, RYR1, RYR2, RYR3, SALL4, SAMD12, SAMD9, SAMHD1, SARDH, SARS1, SASS6, SAT1, SATB1, SATB2, SC5D, SCAF4, SCAPER, SCARB2, SCLT1, SCN10A, SCN11A, SCN1A, SCN1B, SCN2A, SCN3A, SCN4B, SCN5A, SCN8A, SCN9A, SCO2, SCYL2, SDCCAG8, SDHA, SDHAF1, SDHB, SDHC, SDHD, SEC23B, SEC24C, SEC31A, SELENOI, SEMA3A, SEMA4A, SEMA6B, SEPSECS, SERAC1, SERPINI1, SET, SETBP1, SETD1A, SETD1B, SETD2, SETD5, SGPL1, SGSH, SH2B1, SHANK3, SHH, SHQ1, SHROOM4, SIK1, SIK3, SIM1, SIN3A, SIN3B, SIX3, SKI, SLC12A1, SLC12A3, SLC12A5, SLC12A6, SLC13A3, SLC13A5, SLC16A1, SLC16A2, SLC17A5, SLC18A3, SLC19A1, SLC19A2, SLC19A3, SLC1A2, SLC1A3, SLC1A4, SLC20A2, SLC22A5, SLC25A1, SLC25A10, SLC25A12, SLC25A13, SLC25A15, SLC25A19, SLC25A20, SLC25A22, SLC25A36, SLC25A4, SLC25A42, SLC25A46, SLC2A1, SLC2A3, SLC31A1, SLC32A1, SLC33A1, SLC35A1, SLC35A2, SLC35A3, SLC35C1, SLC37A4, SLC38A3, SLC39A8, SLC3A1, SLC44A1, SLC45A1, SLC46A1, SLC4A10, SLC5A6, SLC5A7, SLC6A1, SLC6A19, SLC6A5, SLC6A8, SLC7A6OS, SLC9A6, SLITRK2, SMAD2, SMAD4, SMARCA2, SMARCA4, SMARCAL1, SMARCB1, SMARCC2, SMARCD1, SMARCE1, SMC1A, SMC3, SMC5, SMCHD1, SMG9, SMO, SMPD1, SMPD4, SMS, SNAP25, SNAP29, SNF8, SNIP1, SNORD115-1, SNORD116-1, SNORD118, SNRPN, SNTA1, SNX14, SON, SORL1, SOX10, SOX11, SOX2, SOX3, SOX4, SOX5, SOX9, SP110, SPAST, SPATA7, SPEN, SPG11, SPINK5, SPOP, SPP1, SPR, SPRED1, SPRY4, SPTAN1, SPTBN1, SPTBN4, SPTSSA, SQOR, SQSTM1, SRCAP, SRD5A3, SRPX2, SSR4, ST3GAL3, ST3GAL5, STAG1, STAG2, STAMBP, STAR, STARD7, STAT2, STAT3, STAT4, STEEP1, STIL, STRADA, STS, STT3A, STT3B, STUB1, STX11, STX16, STX1B, STXBP1, STXBP2, SUCLA2, SUCLG1, SUFU, SUMF1, SUOX, SUPT16H, SURF1, SV2A, SVBP, SYN1, SYNGAP1, SYNJ1, SYP, SYT2, SZT2, TACR3, TAF1, TAF4, TAF6, TANC2,

	TANGO2, TAOK1, TARS1, TARS2, TASP1, TAT, TBC1D20, TBC1D23, TBC1D24, TBC1D2B, TBCD, TBCE, TBCK, TBK1, TBL1XR1, TBP, TBR1, TBX1, TBX19, TBX5, TCEAL1, TCF20, TCF4, TCIRG1, TCTN1, TCTN2, TCTN3, TDP1, TDP2, TECPR2, TEFM, TELO2, TERT, TET3, TFAP2A, TFE3, TGDS, TGFB1, TGFBR2, TGFBR3, TGIF1, THOC2, THPO, THSD1, THUMPD1, TIAM1, TICAM1, TIMM50, TIMMDC1, TK2, TLK2, TLR3, TLR4, TLR7, TMEM106B, TMEM138, TMEM147, TMEM163, TMEM165, TMEM216, TMEM218, TMEM222, TMEM231, TMEM237, TMEM67, TMEM70, TMEM94, TMLHE, TMTC3, TMX2, TNFAIP3, TNFRSF11A, TNFRSF11B, TNFSF11, TNFSF4, TNIP1, TNPO2, TNRC6B, TOE1, TOGARAM1, TOMM40, TOPORS, TP53, TP53RK, TP73, TPK1, TPM2, TPP1, TPRKB, TRAF3, TRAF3IP2, TRAF7, TRAK1, TRAPPC10, TRAPPC11, TRAPPC12, TRAPPC2L, TRAPPC4, TRAPPC6B, TRAPPC9, TRDN, TREM2, TREX1, TRIM32, TRIM71, TRIM8, TRIO, TRIP12, TRIP13, TRIT1, TRMT1, TRMT10A, TRNT1, TRPM3, TRPM6, TRPV3, TRRAP, TSC1, TSC2, TSEN15, TSEN2, TSEN34, TSEN54, TSFM, TSPOAP1, TTC5, TTC8, TTI1, TTR, TUBA1A, TUBA8, TUBB2A, TUBB2B, TUBB3, TUBB4A, TUBB4B, TUBG1, TUBGCP4, TUBGCP6, TULP1, TWIST1, TWNK, TXN2, TXNRD2, TYROBP, U2AF2, UBA5, UBAC2, UBAP1, UBAP2L, UBE2A, UBE2L3, UBE3A, UBE4A, UBE4B, UBR7, UBTF, UCP2, UFC1, UFD1, UFM1, UFSP2, UGDH, UGP2, UGT1A1, UNC13D, UNC80, UNC93B1, UPB1, UPF3B, UQCC2, USF3, USP18, USP45, USP7, USP9X, VAMP1, VAMP2, VARS1, VARS2, VCP, VDR, VLDLR, VPS11, VPS13A, VPS13B, VPS13D, VPS16, VPS35L, VPS41, VPS4A, VPS50, VPS51, VPS53, VRK1, WAC, WARS1, WARS2, WASF1, WBP4, WDPCP, WDR11, WDR26, WDR37, WDR4, WDR45, WDR45B, WDR62, WDR73, WDR81, WFS1, WLS, WWOX, XK, XPA, XPC, XPNPEP3, XPR1, YARS1, YEATS2, YIF1B, YIPF5, YME1L1, YRDC, YWHAE, YWHAG, YY1, ZBTB18, ZBTB20, ZBTB7A, ZC4H2, ZDHHC9, ZEB2, ZFP57, ZFTA, ZFX, ZFYVE26, ZIC2, ZMIZ1, ZMYM2, ZMYND11, ZNF142, ZNF292, ZNF423, ZNF526, ZNF592, ZNF668, ZNFX1, ZNHIT3, ZSWIM6
HP:0000939, Osteoporosis	*ABCC9, ADAMTS2, ADAMTSL2, ADCY10, AIP, ALB, ANAPC1, ANTXR2, ARMC5, ASAH1, ASXL1, ASXL2, ATP6V0A1, ATP7A, ATP7B, ATRX, B3GALT6, B3GAT3, BANF1, BAZ1B, BMP1, BMP15, BMP2, BMP6, BNC1, BRAF, BUD23, CALCR, CANT1, CAVIN1, CBL, CBS, CCN6, CDC73, CDH23, CEACAM3, CEACAM6, CFTR, CHD7, CHST3, CLCA4, CLIP2, COL1A1, COL1A2, COL2A1, COL7A1, CPLX1, CTBP1, CTC1, CTCF, CTDP1, CYB5A, CYP11A1, CYP17A1, CYP19A1, CYP27A1, DCTN4, DHX37, DKC1, DKK1, DNAJC30, DPAGT1, DUSP6, EDNRA, EIF2AK3, EIF4H, ELN, ERCC6, ESR1, ESR2, FAT4, FGF17, FGF8, FGFR1, FKBP10, FKBP6, FLRT3, FOXA2, FSHR, G6PC1, GALNS, GALT, GATA1, GATA4, GBA1, GCLC, GCM2, GK, GLB1, GLI2, GNAS, GNPTAB, GNRH1, GNRHR, GORAB, GPAA1, GPR35, GSTM3, GTF2I, GTF2IRD1, GTF2IRD2, HAMP, HBB, HERC2, HESX1, HFE, HJV, HLA-DQA1, HLA-DQB1, HMOX1, HPGD, HS6ST1, HSD17B4, HSD3B7, HSPG2, HTRA1, IFIH1, IFT122, IFT43, IFT52, IGF1, IL12A, IL12RB1, IL17RD, IPW, IRF5, KCNJ8, KCNN4, KDELR2, KDM1A, KISS1, KISS1R, KIT, LAMA3, LAMB3, LAMC2, LARS2, LETM1, LHX4, LIFR, LIMK1,*

	LMNA, LMX1B, LRP5, LRP6, MAGEL2, MALT1, MAP3K1, MED12, MEN1, METTL27, MIF, MKRN3, MLXIPL, MMEL1, MMP1, MMP14, MMP2, MRPS22, MSH4, MST1, MTRR, NCF1, NDN, NDUFAF1, NELFA, NF1, NFIX, NGLY1, NHERF1, NHLH2, NHP2, NOP10, NOTCH2, NPAP1, NPM1, NR0B1, NR3C1, NR5A1, NSD2, NSMF, NUP107, OCA2, OTX2, PARN, PCCA, PCCB, PDE11A, PDGFRB, PDLIM4, PEX12, PHKA2, PHKB, PHKG2, PIGG, PIGT, PLOD1, PLOD2, PMM2, POF1B, POLD1, POLG, POLG2, POLR3H, POU1F1, POU2AF1, PPIB, PRDM5, PRG4, PRKACA, PRKAR1A, PRLR, PROK2, PROKR2, PROP1, PSMC3IP, PSTPIP1, PWAR1, PWRN1, PYCR1, PYGL, RAB3GAP1, RECQL4, RFC2, RIN2, RNU4-2, RNU4ATAC, RNU7-1, RPL10, RPL11, RRM2B, RTEL1, RUNX1, RUNX2, SATB2, SC5D, SCARB2, SCN4A, SEMA4D, SERPINA1, SGMS2, SH3PXD2B, SIM1, SLC11A1, SLC25A19, SLC25A4, SLC26A9, SLC34A1, SLC37A4, SLC6A14, SLC7A7, SLC9A3, SLC9A6, SLCO2A1, SMAD3, SMPD1, SMS, SNORD115-1, SNORD116-1, SNRPN, SOX3, SOX9, SP7, SPARC, SPIB, SPIDR, SPRTN, SPRY4, SRC, SRSF2, SRY, STAT1, STAT3, STAT6, STX1A, TAC3, TACR3, TBCK, TBL2, TCF12, TCF4, TENT5A, TERC, TERT, TET2, TGFB1, TGFBR2, TINF2, TMEM165, TMEM270, TMEM38B, TMEM67, TNFRSF11A, TNFRSF11B, TNFSF15, TNPO3, TP53, TRAPPC2, TRIP11, TRMT10A, TRPV4, TWNK, TYMS, UROD, UROS, USB1, USP48, USP8, VAMP7, VPS37D, VPS53, WDR11, WDR19, WDR35, WNK3, WNT1, WNT3A, WRAP53, WRN, WT1, WWOX, XYLT2, ZBTB20, ZFPM2, ZNF469, ZSWIM7
